# Managing hardware-related metal artifacts in MRI: current and evolving techniques

**DOI:** 10.1007/s00256-024-04624-4

**Published:** 2024-02-21

**Authors:** Georg C. Feuerriegel, Reto Sutter

**Affiliations:** Department of Radiology, Balgrist University Hospital, Faculty of Medicine, University of Zurich, Forchstrasse 340, 8008 Zurich, Switzerland

**Keywords:** Magnetic resonance imaging, Arthroplasty, Artifacts, Joint prosthesis

## Abstract

Magnetic resonance imaging (MRI) around metal implants has been challenging due to magnetic susceptibility differences between metal implants and adjacent tissues, resulting in image signal loss, geometric distortion, and loss of fat suppression. These artifacts can compromise the diagnostic accuracy and the evaluation of surrounding anatomical structures. As the prevalence of total joint replacements continues to increase in our aging society, there is a need for proper radiological assessment of tissues around metal implants to aid clinical decision-making in the management of post-operative complaints and complications. Various techniques for reducing metal artifacts in musculoskeletal imaging have been explored in recent years. One approach focuses on improving hardware components. High-density multi-channel radiofrequency (RF) coils, parallel imaging techniques, and gradient warping correction enable signal enhancement, image acquisition acceleration, and geometric distortion minimization. In addition, the use of susceptibility-matched implants and low-field MRI helps to reduce magnetic susceptibility differences. The second approach focuses on metal artifact reduction sequences such as view-angle tilting (VAT) and slice-encoding for metal artifact correction (SEMAC). Iterative reconstruction algorithms, deep learning approaches, and post-processing techniques are used to estimate and correct artifact-related errors in reconstructed images. This article reviews recent developments in clinically applicable metal artifact reduction techniques as well as advances in MR hardware. The review provides a better understanding of the basic principles and techniques, as well as an awareness of their limitations, allowing for a more reasoned application of these methods in clinical settings.

## Introduction

The prevalence of total joint replacements is steadily increasing each year, mainly due to the aging population and the increased demands on the physical abilities of the elderly. A total of 743,327 knee and hip replacements were performed in the USA in the year 2019, and an increase of approximately 139–176% is estimated for the year 2040 creating a need for appropriate diagnostic imaging tools around metal implants [1]. There are several imaging modalities that can be used to assess and diagnose postoperative complications after joint replacement, including ultrasound, computed tomography, and radiography. However, because of its good soft tissue contrast, magnetic resonance imaging (MRI) has proved invaluable in diagnosing postoperative complications following joint replacement [2, 3]. In postoperative patients with complaints after joint replacements, common suspected abnormalities include infection and abscess formation, pseudotumour formation, nonunion, aseptic loosening, fracture, and soft tissue abnormalities. MRI around metal implants remains challenging, but because of technological advances in recent years, MRI of metal implants is a major focus in musculoskeletal imaging. Improvements in equipment hardware such as multi-channel coils, as well as the optimization of dedicated MR protocols to reduce metal artifacts, have helped to address a variety of clinical questions. In this review, the physical background of metal artifact formation and the influencing factors are discussed. Furthermore, different strategies for metal artifact reduction, including hardware- and software-based approaches, are presented and the advantages and disadvantages of these techniques are discussed. As a special focus, this review includes the recent advantages of low-field MRI and deep learning–based image reconstruction for metal artifact reduction.

## Artifact formation on MRI—independent/uncontrolled factors

MRI artifacts are mainly caused by variations in the resonance frequency, which are due to differences in the magnetic susceptibility between metal implants and the surrounding tissue. The magnetic susceptibility of a substance describes how it is magnetized in a given magnetic field [4]. For example, ferromagnetic materials such as iron, cobalt, or nickel cause a greater change in the local magnetic field than paramagnetic materials such as titanium and gadolinium. The constant, homogeneous magnetic field of any MR scanner is called the B_0_ field and is used to polarize the spins, creating magnetization. In contrast, the B_1_ field is a high-frequency energy field applied perpendicularly to the B_0_ field to perturb the net magnetization, such as excitation or inversion pulses. Achieving a linear, homogeneous main magnetic field B_0_ is crucial for accurate signal encoding and image reconstruction in MRI. The sharp magnetic susceptibility transitions of metal implants to the surrounding paramagnetic soft tissue cause local inhomogeneities in the B_0_ field. The inhomogeneous B_0_ field during MRI acquisition results in three main effects: signal loss and pile-up, geometric distortion, and fat suppression failure [5]. MRI artifacts in the presence of metal implants are most severe near the implant and distant structures can be imaged with fewer limitations. It is therefore important to assess whether the region of interest will be affected by artifacts prior to imaging. In patients with, e.g., unilateral hip replacement, the contralateral hip can be assessed without dedicated sequences.

### Signal loss and pile-up

**Fig. 1:**
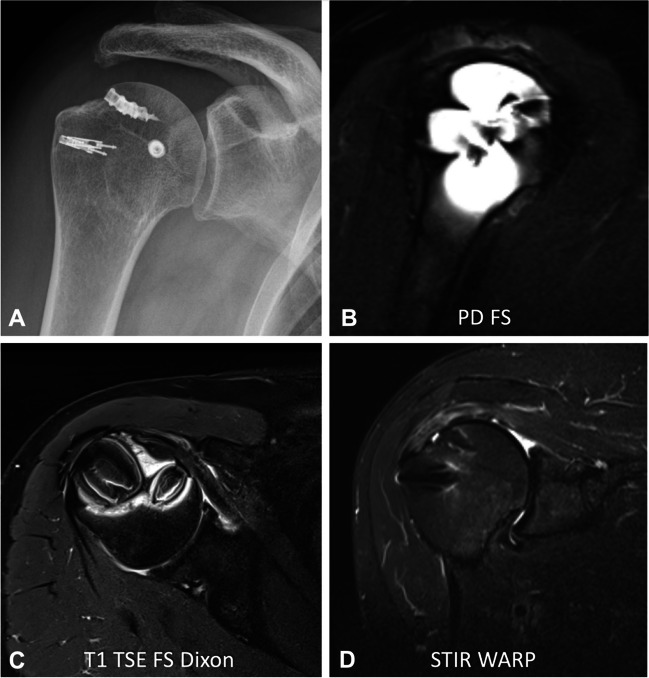
A 61-year-old patient after rotator cuff repair. The anteroposterior radiograph (**A**) shows four suture anchors in the humeral head made of titanium. The standard sagittal fat-saturated proton density (PD)-weighted image (**B**) shows the typical four-leaf clover artifact caused by the ferromagnetic metal implants, which interferes with the diagnostic evaluation of the humeral head and the insertion of the rotator cuff tendons. The axial T1-weighted Dixon MR sequence (**C**) demonstrates typical artifacts caused by the ferromagnetic metal implants in Dixon sequences. A dedicated short-tau inversion recovery (STIR) turbo spin echo sequence with optimized inversion pulse (STIR WARP) (**D**) shows significantly less artifacts with adequate fat suppression, allowing assessment of the integrity of the rotator cuff insertion. The images were acquired at 1.5 T with following sequence parameters: PD FS—echo time (TE) 58 ms, repetition time (TR) 3370 ms, receiver bandwidth 435 Hz/pixels; T1 TSE FS Dixon—TE 36 ms, TR 3620 ms, receiver bandwidth 250 Hz/pixel; STIR WARP—TE 47 ms, TR 4000 ms, receiver bandwidth 300 Hz/pixel, inversion time (IT)170 ms

Changes in the local magnetic field due to metallic implants result in rapid dephasing and incoherence of the spins within a single voxel [6, 7]. The resonance of the protons is shifted out of the bandwidth of the radiofrequency pulse, resulting in signal loss, manifested as black spots due to the absence of signal at the expected location, or signal pile-up due to increased signal at another location. Ferromagnetic implants exhibit a typical four-leaf clover pattern where the implant acts as a dipole, causing inhomogeneities with both suppression and enhancement of the local B_0_ magnetic field (Fig. 1).

### Geometric distortions

**Fig. 2:**
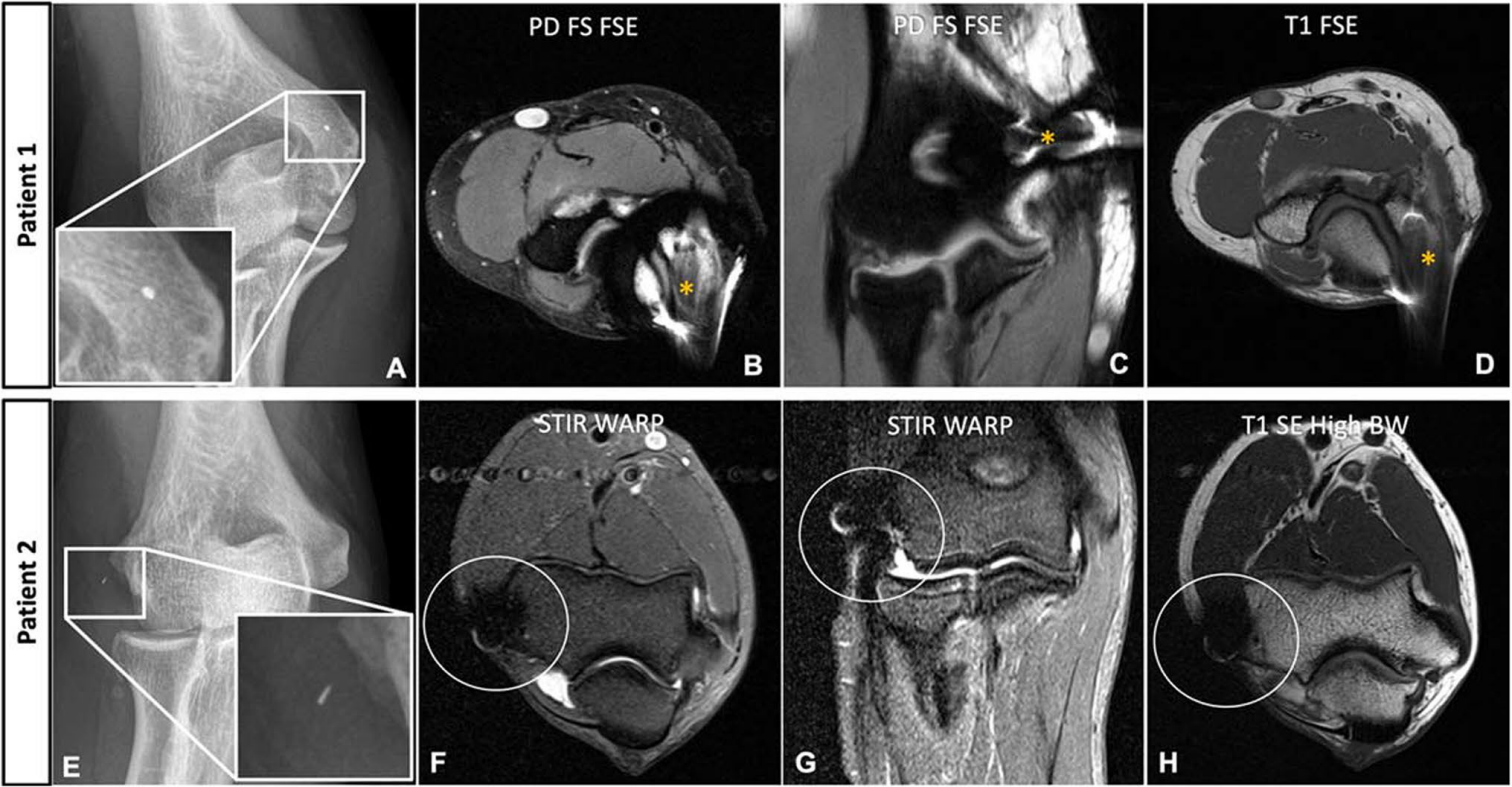
Signal pile-up and geometric distortion caused by a residual metal fragment after removal of orthopedic hardware from the distal humerus in a 38-year-old patient (patient 1, **A**–**D**) and a 41-year-old patient (patient 2, **E**–**H**). The MRI of the first patient was acquired with standard proton density turbo spin echo (PD TSE) sequences with fat saturation in axial (**B**) and coronal (**C**) orientation, as well as a standard T1-weighted TSE sequence in axial orientation (**D**). Note the significantly increased signal pile-up and geometric distortion in the standard sequences (asterisks). Additionally, a failure of fat suppression can be seen on the coronal PD TSE sequence around the metal fragment (**C**). Patient 2 was examined with dedicated metal artifact reduction sequences including an axial and sagittal short-tau inversion recovery (STIR) TSE sequence with optimized inversion pulse (STIR WARP) (**F**, **G**) and a high bandwidth (BW) T1-weighted TSE sequence. Note that only localized artifacts are seen around the metal fragment (white circle). The severity of artifacts is due to the ferromagnetic properties of the broken-off metal fragments. The images were acquired at 1.5 T with following sequence parameters: transverse PD FS—echo time (TE) 26 ms, repetition time (TR) 2902 ms, receiver bandwidth 168 Hz/pixel; coronal PD FS—TE 24 ms, TR 2341 ms, receiver bandwidth 104 Hz/pixel; T1 FSE—TE 11 ms, TR 603 ms, receiver bandwidth 107 Hz/pixel; transverse STIR WARP—TE 41 ms, TR 3000 ms, receiver bandwidth 360 Hz/pixel, inversion time (TI) 130 ms; coronal STIR WARP—TE 22 ms, TR 3000 ms, receiver bandwidth 400 Hz/pixel, IT 130 ms; T1 SE High BW—TE 11 ms, TR 400 ms, receiver bandwidth 270 Hz/pixel

The generation of an MR image relies on the precise localization of each tissue voxel through the application of position-dependent gradient fields during both slice selection and readout. Metal-induced variations in the B_0_ field lead to errors in the position of the selected slice, ultimately resulting in a change in the precession frequency of the affected spins [8]. As a result, spins outside the selected slice are unintentionally excited, and the selected slice is distorted by data from adjacent slices, known as through-plane or “potato chip” artifacts. Furthermore, during the readout phase, pixels become improperly aligned with the wrong positions along the frequency-encoding (readout) direction [4]. Ideally, the encoding gradient should be linear, but local field gradients can distort it so that it becomes curvilinear, with the result that the position of a voxel’s data along that axis is misregistered (Fig. 2). However, since local field gradients do not affect the phase of precession, the phase encoding direction is insensitive to in-plane artifacts [8]

### Failed fat suppression

**Fig. 3:**
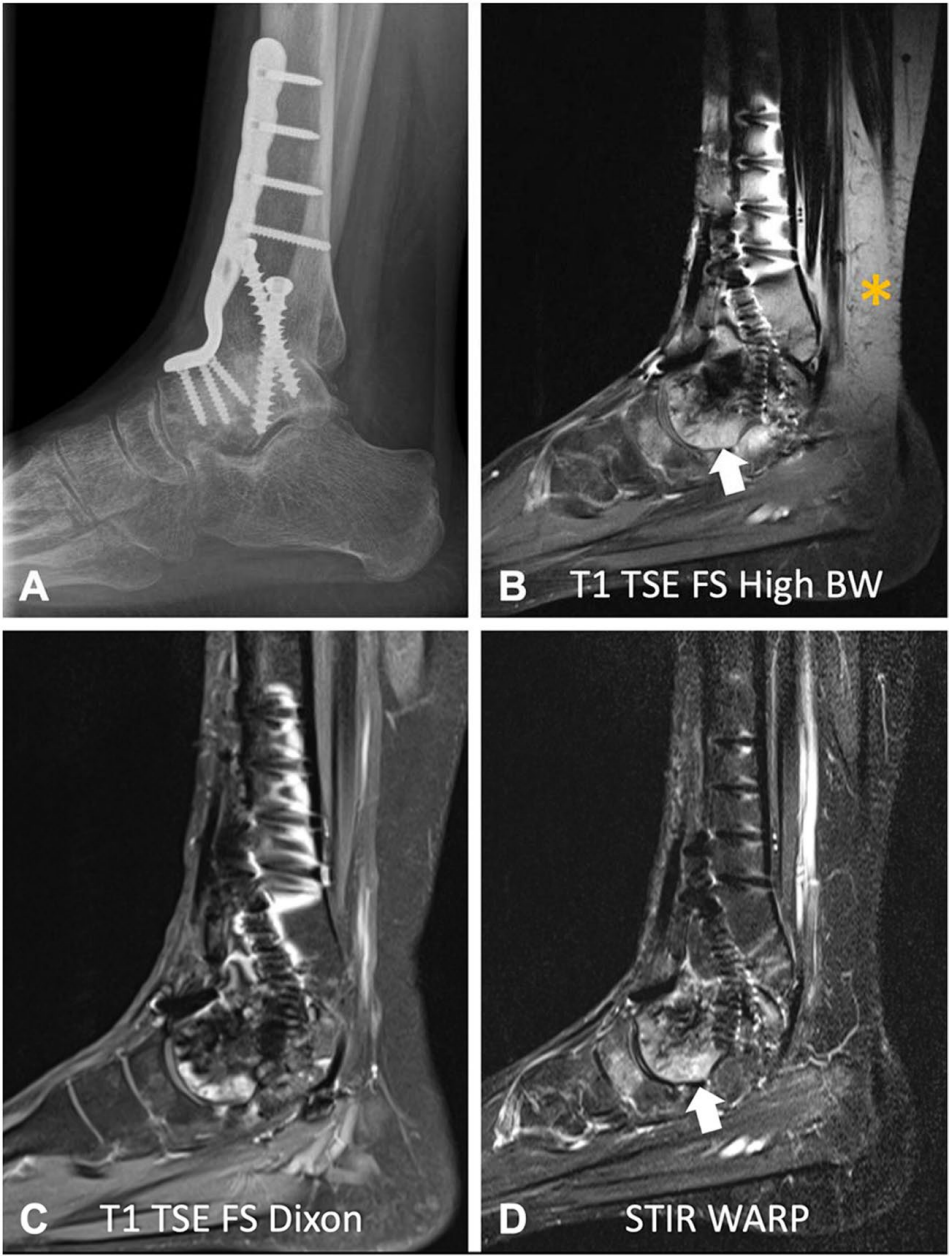
A 54-year-old patient after arthrodesis of the upper ankle joint following curettage and plombage of an aneurysmatic bone cyst with titanium screws and plates. The patient suffered from recurrent ankle pain on movement. The lateral radiograph (**A**) shows several types of metal implants in different orientations. The high bandwidth (BW) sagittal T1-weighted fat-suppressed turbo spin echo (TSE) sequence (**B**, bandwidth 372 Hz/pixel) shows severe signal accumulation and geometric distortion with failure of fat saturation (asterisk). Note also that the failure of fat suppression does not allow a reliable detection of the bone marrow edema in the talus (arrow), which was most likely the source of the ankle pain, as the signal in the talus looks similar to the signal in the distal tibia. The sagittal T1-weighted Dixon sequence (**C**) provides adequate overall fat suppression, but significant focal artifacts remain around the implants. A sagittal short tau inversion recovery (STIR) turbo spin echo sequence (**D**) with optimized inversion pulse (STIR WARP) demonstrated the least artifacts and provided adequate fat suppression, visualizing the bone marrow edema of the talus head (arrow). The images were acquired at 1.5 T with following sequence parameters: T1 TSE FS High BW—echo time (TE) 10 ms, repetition time (TR) 631 ms, receiver bandwidth 372 Hz/pixel; T1 TSE FS Dixon—TE 12 ms, TR 655 ms, receiver bandwidth 248 Hz/pixel; STIR WARP—TE 48 ms, TR 4000 ms, receiver bandwidth 385 Hz/pixel, inversion time (IT) 160 ms

Chemical-shift-selective fat suppression relies on the distinct resonance frequencies of fat and water protons. For effective fat saturation, a short-duration RF pulse tuned to the RF of fat is applied before the MR image acquisition. This causes the fat signal to be selectively saturated (nulled) while the water signal remains unaffected [8, 9]. B_0_ inhomogeneities induced by metal implants shift the fat resonance peak out of the frequency range targeted by the saturation pulse, resulting in failure of fat suppression or even cause saturation of the water signal [6] (Fig. 3).

## Metal artifact reduction in MRI—basic parameter changes

Over the past decade, several approaches have been proposed to reduce metal artifact in MRI, of which three main approaches have shown the most promise. The first approach is to improve the interaction of the metal implant with the MR hardware. The second is to adjust and adapt conventional MR sequence parameters and the third is to apply advanced metal artifact reduction techniques such as multispectral imaging, advanced reconstruction techniques, post-processing, and deep learning–based techniques.

## MR hardware

### Field strength

The severity of metal artifacts depends on magnetic field strength and intensifies with increasing field strength due to the linearly increasing magnetic field differences caused by the susceptibility difference between the metal implant and the surrounding soft tissue [5, 6, 10]. Consequently, imaging at a lower field strength of 0.55 T produces fewer susceptibility artifacts, increasing linearly to the most artifacts at 7.0 T. However, the use of low-field MRI has the disadvantage of a lower signal-to-noise ratio (SNR) and lower image resolution, while increased specific absorption rates (SAR) are a significant limitation at higher field strengths [5, 11, 12]. Metal artifact reduction techniques can be applied regardless of magnetic field strength, but if available, a 1.5-T scanner is preferred for imaging patients with metal implants, as it offers the best trade-off between reduced magnetic field differences and higher SNR [13].

### Influence of the implant

The main reasons for artifacts in MR imaging with metal implants are due to differences in the magnetic susceptibility between metal implants and the surrounding tissue. Ideally, implants would exhibit the same magnetic susceptibility as the surrounding tissue in order to reduce both in-plane and through-plane artifacts. Signal void (dark) and pile-up (bright) artifacts from metal occur in multiple directions. In-plane refers to artifacts occurring within the imaging slice and through-plane refers to artifacts displaced from one slice to others. Titanium is now one of the most commonly used materials for metal implants which causes significantly less artifacts compared to stainless steel or cobalt-chromium [14–18]. Current research is focusing on further reducing the magnetic susceptibility of metal implants by synthesizing aluminum-free titanium composite materials or carbon-fiber-reinforced polymers and biodegradable magnesium alloys [19–22].

### Positioning

In general, metal implants produce the least artifacts when the long axis is aligned with the magnetic field B_0_ [23–25]. However, manual repositioning of the metal is often not possible given the body region of the metal implant, the region to be imaged, and the architecture of the MRI magnet. In these cases, swapping the phase-encoding direction and the frequency-encoding direction can help to reduce artifacts by changing the direction in which the metal artifacts propagate [26]. Therefore, due to the lack of long axis and variable orientation, imaging of complex implants may be more difficult [11].

## Sequence optimization

**Fig. 4:**
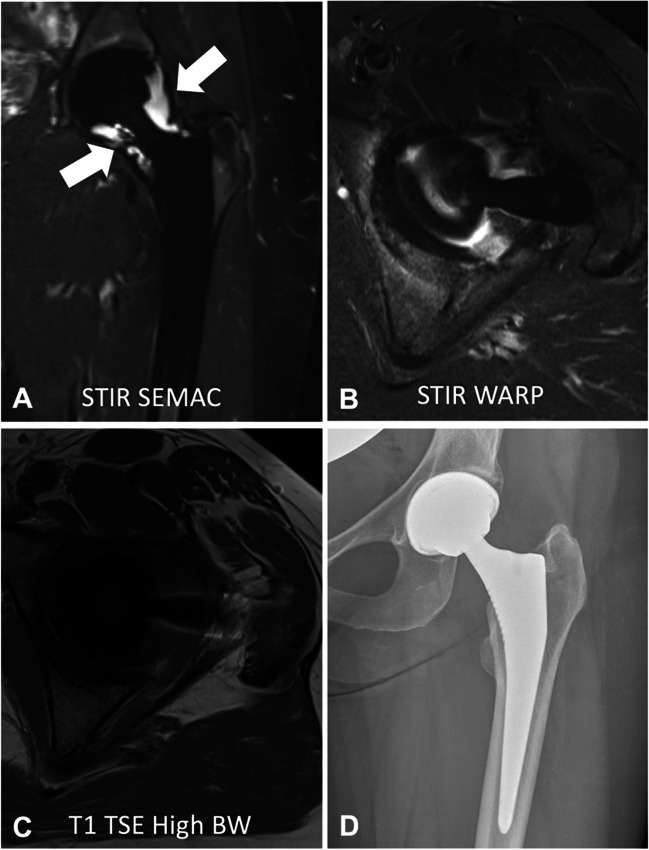
Advanced metal artifact reduction imaging of the hip after total joint arthroplasty in a 67-year-old patient (**A**–**D**). The implant is made of a titanium alloy. Adequate metal artifact suppression is demonstrated with a dedicated coronal slice encoding for metal artifact correction (SEMAC) turbo spin echo sequence, allowing diagnosis of the joint effusion surrounding the head of the femoral prosthesis (**A**, arrows). The SEMAC sequence shows the least artifacts compared to the axial short-tau inversion recovery (STIR) turbo spin echo sequence (**B**) with optimized inversion pulse (STIR WARP) and a high bandwidth (BW) T1-weighted turbo spin echo sequence (**C**) (bandwidth 425 Hz/pixel). The conventional radiograph (**D**) demonstrates normal fit of the prosthesis without signs of loosening. The images were acquired at 1.5 T with following sequence parameters: STIR SEMAC WARP—echo time (TE) 36 ms, repetition time (TR) 4220 ms, receiver bandwidth 500 Hz/pixel, inversion time (IT) 160 ms; transverse STIR WARP—TE 31 ms, TR 3830 ms, receiver bandwidth 450 Hz/pixel, IT 150 ms; T1 TSE High BW—TE 9 ms, TR 600 ms, receiver bandwidth 425 Hz/pixel

**Fig. 5:**
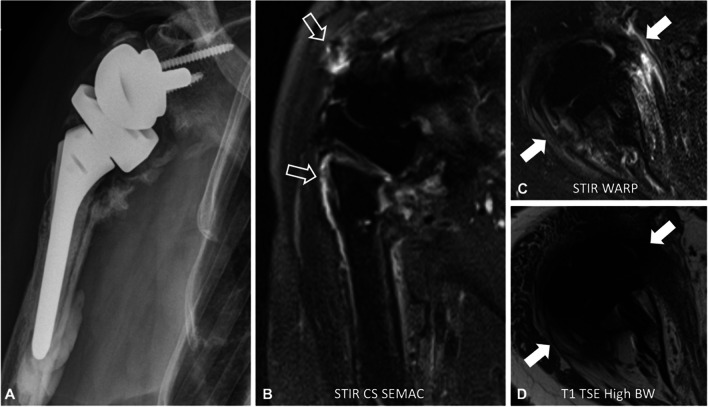
A 61-year-old patient with shoulder arthroplasty using implants made of titanium alloys. The radiograph (**A**) shows the large and complex implant with signs of loosening between the cement and the bone of the proximal humerus. The coronal short tau inversion recovery (STIR) turbo spin echo sequence (**B**) combined with compressed sensing (CS) and slice encoding for metal artifact correction (SEMAC) demonstrate only little artifacts within an acceptable acquisition time of 6:31 min and allows assessment of the fluid and increased signal around the implant indicating low-grade inflammation (outline arrows). After administration of intravenous contrast, the axial short-tau inversion recovery (STIR) turbo spin echo sequence (**C**) with optimized inversion pulse (STIR WARP) and a high bandwidth (BW) T1-weighted turbo spin echo sequence (**D**) (bandwidth 480 Hz/pixel) show a corresponding onion-like enhancement of the surrounding tissue (**C**, white arrows). Joint aspiration proved a low-grade infect with *Cutibacterium acnes*. The images were acquired at 1.5 T with following sequence parameters: STIR SEMAC—echo time (TE) 44 ms, repetition time (TR) 4500 ms, receiver bandwidth 780 Hz/pixel, inversion time (IT) 145 ms; STIR WARP—TE 33 ms, TR 4250 ms, receiver bandwidth 390 Hz/pixel, TI 160 ms; T1 TSE High BW—TE 10 ms, TR 648 ms, receiver bandwidth 480 Hz/pixels

There are several basic methods to improve image quality and reduce metal artifacts when imaging metal implants. Basic sequence optimization steps include smaller nominal voxel size encoding (thinner slices, large image matrix), high receiver and RF pulse bandwidth, shorter echo times, and changing the direction of frequency and phase encoding directions. In addition, acquisition of fast spin-echo and STIR sequences rather than single spin-echo and gradient-echo sequences further reduces image artifact. However, changing the sequence parameters often leads to a reduction in SNR and the residual metal artifacts may be substantial, which is why advanced metal artifact reduction techniques such as view angle tilting (VAT), slice encoding for metal artifact correction (SEMAC), and multiacquisition variable-resonance image combination (MAVRIC) have been developed to further improve image quality (Figs. 4 and 5).

### Choosing the right sequence

In general, fast spin-echo and turbo spin-echo sequences are preferable to gradient-echo sequences due to the rapid intravoxel dephasing of spins in gradient-echo sequences resulting in signal loss and increased artifacts [27, 28]. Spin-echo sequences exhibit a refocusing pulse applied at half of the time interval between the initial 90° flip and the echo which is able to correct in part for focal signal inhomogeneities such as metal artifacts. In contrast, gradient-echo sequences do not have a refocusing pulse. When acquiring 3-dimensional (3D) TSE sequences, it is important to remember that the slice-encoding direction is also phase-encoded and therefore non-selective or slice-selective excitation must be chosen [5, 29]. In order to reduce the potential for spatial mis-selection and through-plane distortion, non-selective excitation should be used, as it does not require a slice selection encoding gradient [30]. However, any spins outside the excitation bandwidth of the RF pulse will not be excited and these regions will appear dark. Therefore, sufficiently high RF bandwidths are also recommended for 3D non-selective sequences [31].

### Receiver bandwidth

Increasing the receiver bandwidth is one of the most effective and simple measures to reduce in-plane artifacts. A higher bandwidth corresponds to a stronger gradient, which reduces signal mis-registration in the in-plane frequency encoding direction [32, 33]. Common receiver bandwidths for imaging of the knee joint with and without metal implants are illustrated in Tables 1 and 2. However, higher bandwidths require strong gradients, which is often a limitation. In addition, the receiver bandwidth is inversely related to the SNR: When the bandwidth increases, the SNR decreases. This can be compensated for by increasing the number of excitations, which prolongs the scan time and may also lead to an increase in the total RF energy applied to the patient being examined, which can be another limitation, particularly at higher field strengths [34, 35].

**Table 1:** Sample protocol at 1.5-T MRI for patients with knee prostheses

Knee pulse sequences	Transverse STIR	Coronal STIR	Sagittal IM	Sagittal STIR	Coronal T1
MARS technique	–	SEMAC CS	SEMAC CS	SEMAC CS	–
Echo time (ms)	37	39	35	39	8.3
Repetition time (ms)	5220	4000	4500	4020	600
Acquisition matrix (mm)	752 × 736	320 × 320	384 × 384	320 × 320	832 × 832
Slice thickness (mm)	3.5	4	3.5	3.5	3
Flip angle (°)	135	180	180	180	135
FOV (mm)	183 × 180	200 × 200	199 × 199	200 × 200	200 × 200
Bandwidth (Hz/pixel (kHz))	412	539	521	539	523
Inversion time (ms)	160	160	–	160	–
Averages	2	1	1	1	2
Turbo factor	9	11	19	11	4
Number of slices	46	30	32	32	32
Phase-encoding steps	263	224	307	224	333
Slice-encoding steps	–	15	12	12	–
Direction	R > > L	R > > L	A > > P	H > > F	H > > F
Acquisition time (min)	2:33	5:18	3:47	4:27	2:10

*MARS* metal artifact reduction sequences, *STIR* short tau inversion recovery, *CS* compressed sensing, *IM* intermediate weighted

**Table 2:** Sample protocol of the knee at 3-T MRI for patients without metal implants

Knee pulse sequences	Coronal STIR	Sagittal IM	Sagittal IM	Transverse IM	Coronal T1
Echo time (ms)	40	31	32	41	10
Repetition time (ms)	5000	3250	2810	4390	550
Acquisition matrix (mm)	384 × 269	448 × 314	512 × 358	418 × 314	512 × 410
Slice thickness (mm)	3	3	3	2.5	3
Flip angle (°)	160	135	135	180	135
FOV (mm)	159 × 159	160 × 160	199 × 199	149 × 149	159 × 159
Bandwidth (Hz/pixel (kHz))	181	180	181	180	244
Inversion time (ms)	210	–	–	–	–
Averages	2	1	1	1	1
Turbo factor	9	7	7	7	3
Number of slices	28	31	32	40	28
Phase-encoding steps	269	314	358	314	410
Acquisition time (min)	1:45	1:18	1:18	1:19	0:58

*STIR* short tau inversion recovery, *IM* intermediate weighted

### Matrix sizes and slice thickness

Other simple sequence adjustments include increasing the matrix size and decreasing the slice thickness. Increasing the matrix size results in a smaller encoded voxel size and therefore reduces in-plane distortion and intra-voxel dephasing [6, 24, 36]. It is important to note that matrix increase is most efficient when performed in the frequency-encoding direction. For smaller slice thicknesses, stronger slice selection gradients are required, which reduce through-plane artifacts. In addition, the spread of frequency differences within the smaller voxel may be reduced [7]. However, a slice thickness of less than 3 mm is associated with increased scan time, which can be compensated for by increasing the interslice spacing, but at the expense of tissue coverage. Thinner slices are also associated with decreased SNR and increased SAR; therefore, a slice thickness of 3 to 4 mm is considered adequate in clinical routine.

### Frequency and phase encoding directions

Another simple adjustment to reduce metal implant artifacts is to change the direction of the phase and frequency encoding. In-plane mis-registrations usually occur along the frequency encoding direction, and changing the direction is often a simple fix to shift metal artifacts away from the region of interest [7, 37].

### Fat suppression techniques

Several fat suppression techniques are available in clinical routine, the most common being chemical shift–based techniques such as spectral (selective) fat saturation and Dixon imaging, and inversion-based techniques such as short-tau inversion recovery (STIR) [38, 39]. Dixon methods such as IDEAL (Iterative Decomposition of Water and Fat with Echo Asymmetry and Least Squares Estimation) offer better fat suppression in the presence of metallic implants than standard fast spin echo sequences and, unlike STIR imaging, allow the use of contrast-enhanced MR imaging, providing better diagnostic performance in the assessment of post-operative infection [40–42]. However, compared to STIR sequences, chemical shift–based techniques are more likely to fail due to their strong dependence on B_0_ and B_1_ homogeneities. In general, STIR sequences are less dependent on B_0_ and B_1_ inhomogeneities, making them the fat suppression sequences of choice for imaging near metal implants [5]. STIR is based on the different T1 relaxation times and recovery of longitudinal magnetization after application of a 180° inversion pulse up to the application of a 90° excitation pulse which reverses the intravoxel dephasing. This inversion time (TI) can also be used to modulate fat saturation [43]. As a limitation, STIR sequences generally offer a lower SNR compared to chemical shift–based fat suppression at equivalent acquisition times and are also limited in the use of post contrast imaging because enhancing tissues may have similarly short T1 relaxation times as fat and may also be suppressed [44].

## Evolving techniques

### View angle tilting

As an approach to correct in-plane distortions, VAT has been proposed by Cho et al. [45]. In this approach, a superimposed slice selection gradient is applied simultaneously with the readout gradient, causing a tilting of the readout encoding dimension towards the slice selection dimension. Consequently, all excited spins in the targeted slice process at the same frequency, eliminating any off-resonance induced shift along the readout direction. Although VAT does not increase scan time, it introduces some blurring, which can be diminished by reducing slice thickness [46]. As VAT depends on the ratio of slice gradient to readout gradient, the best artifact reduction is achieved at lower bandwidths. However, it should be noted that VAT does not correct for through-plane artifacts, which is considered to be its main limitation [47].

### Slice encoding for metal artifact correction

Compared to conventional artifact reduction techniques, SEMAC has been shown to be an efficient technique for imaging near metal implants (Figs. 4 and 5). Although based on a 2D sequence where individual slices are excited with an RF pulse, additional phase encoding steps in the slice selection dimension are applied to resolve a larger 3D slab around each slice position. The additional information about the distortion of the slices can then be used to correct for through-plane distortion of the acquired slice and of the adjacent slices [48–50]. The number of additional phase encoding steps can be chosen individually for each scan, depending on the size and complexity of the metal implant and the area of field inhomogeneity. However, increasing the number of steps also increases the scan time, which ultimately leads to a trade-off between the degree of artifact reduction and acquisition times. In literature, it has been proposed that 11 slice-encoding steps (for T2-weighted images) to 19 slice-encoding steps (for STIR and T1-weighted images) might be sufficient for artifact reduction around hip prostheses [5, 51]. The SEMAC technique addresses through-plane artifacts, but it is often implemented with VAT to address in-plane artifacts as well.

### Multiacquisition variable-resonance image combination

MAVRIC has been developed to address metal-induced in-plane and through-plane distortions caused by the field inhomogeneities surrounding the implant [52, 53]. It uses a 3D acquisition technique with a spatially non-selective excitation which—in contrast to gradient-based slice selection—performs phase encoding along two dimensions with reduced distortion. In general, spin precession around metal implants occurs over a wide range of frequencies due to B_0_ inhomogeneities; the bandwidth of a single non-selective RF pulse is not wide enough to cover the full range of off-resonance frequencies and the lack of excitation would result in signal loss [54]. To overcome this problem, MAVRIC acquires spectral bins, which are essentially 3D slabs with slightly different resonance frequencies. The acquired 3D slabs are combined into a composite image using a sum-of-squares or maximum intensity projection scheme. On the down side, MAVRIC lacks slice selectivity which requires time-consuming 3D imaging.

### Hybrid approaches and future developments

Several hybrid approaches have been proposed to overcome some of the limitations of the techniques themselves [55, 56]. One variant combines the slab selectivity of SEMAC with the smooth bin combination and higher SNR implemented by MAVRIC. The so-called MAVRIC SL technique combines the slice-direction phase encoding of SEMAC with the increased spectral coverage and has demonstrated improved image quality and reduced image artifacts when imaging patients with total hip and shoulder prosthesis (Table 3).

**Table 3:** Sample protocol at 1.5-T MRI for patients with shoulder prostheses

Shoulder pulse sequences	Transverse T1	Transverse STIR	Coronal STIR	Sagittal IM
MARS technique	–	–	SEMAC CS	SEMAC CS
Echo time (ms)	6.6	38	44	35
Repetition time (ms)	685	4000	5200	5000
Acquisition matrix (mm)	384 × 384	320 × 320	256 × 256	256 × 256
Slice thickness (mm)	4	4	4	3.5
Flip angle (°)	132	135	140	180
FOV (mm)	180 × 180	180 × 180	200 × 200	200 × 200
Bandwidth (Hz/pixel (kHz))	401	402	539	521
Inversion time (ms)	–	160	160	–
Averages	1	1	1	1
Turbo factor	3	12	11	19
Number of slices	30	36	20	25
Phase-encoding steps	619	299	302	261
Slice-encoding steps	-	12	12	-
Direction	A > > P	A > > P	A > > P	R > > L
Acquisition time (min)	2:47	2:26	5:45	4:12

*MARS* metal artifact reduction sequences, *STIR* short tau inversion recovery, *CS* compressed sensing, *IM* intermediate weighted

In order to reduce the scan time of SEMAC, a combination with off-resonance suppression (ORS) was proposed [57]. In this technique, separate RF bandwidths and gradient strengths of the excitation and refocusing pulses are applied. Therefore, the range of spins which contribute to the image are limited and fewer phase-encoding steps are necessary for SEMAC. This approach limits back-folding artifacts and enables flexibility of scan orientation with the disadvantages of signal voids and loss of homogeneity.

As described above, the large static gradients in the B_0_ field in close proximity to metal implants result in increased intravoxel dephasing and rapid T2* decay. Ultra-short echo time allows imaging of tissues with very short T2 and allows the signal to be acquired before it has dephased [58]. A hybrid technique combining UTE and MAVRIC has been proposed for imaging of hip prosthesis, attempting to combine the advantages of multispectral imaging of MAVRIC and a non-selective 3D UTE [59]. Further research has focused on the development of an externally calibrated parallel imaging technique for three-dimensional multispectral imaging (3D-MSI) using broadband UTE [60]. The technique allows for a significant reduction in scan time while maintaining similar metal artifact reduction as conventional MAVRIC acquisitions (Table 4).

**Table 4:** Sample protocol at 1.5-T MRI for patients with minor surgical implants of the shoulder such as anchors or screws

Shoulder pulse sequences	Coronal IM	Coronal STIR	Sagittal T1	Sagittal STIR	Transversal IM	Transversal STIR
Echo time (ms)	29	35	10	35	29	35
Repetition time (ms)	3210	4000	548	4000	3200	4000
Acquisition matrix (mm)	768 × 768	640 × 640	768 × 768	640 × 640	768 × 768	640 × 640
Slice thickness (mm)	3	3	4	4	3	3
Flip angle (°)	135	135	135	135	135	135
FOV (mm)	159 × 159	160 × 160	160 × 160	160 × 160	159 × 159	160 × 160
Bandwidth (Hz/pixel (kHz))	449	411	407	411	407	401
Inversion time (ms)	–	160	–	160	–	160
Averages	2	2	1	2	2	2
Turbo factor	14	9	3	12	12	11
Number of slices	27	27	29	23	30	28
Phase-encoding steps	307	224	288	224	307	224
Phase encoding direction	F > > H	F > > H	F > > H	H > > F	A > > P	A > > P
Acquisition time (min:s)	1:47	2:54	1:27	2:14	1:58	3:06

*STIR* short tau inversion recovery, *IM* intermediate weighted

**Fig. 6:**
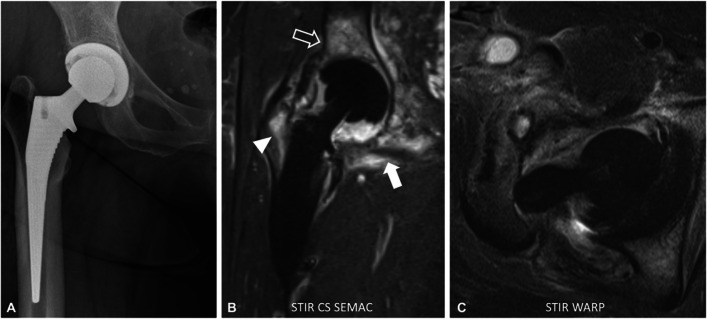
1.5-T MRI of the right hip of a 57-year-old patient 1.5 years after total hip arthroplasty. The radiograph shows a normal position of the implant made of titanium alloy (**A**). However, the coronal short-tau inversion recovery (STIR) turbo spin echo sequence (**B**) combined with compressed sensing (CS) and slice encoding for metal artifact correction (SEMAC) shows severe joint inflammation with increased synovial fluid, extensive bone marrow edema (arrowhead and outline arrow), and strongly hyperintense signal of the surrounding soft tissues (arrow); this imaging pattern is highly suspicious of a periprosthetic joint infection. The axial short-tau inversion recovery (STIR) turbo spin echo sequence (C) with optimized inversion pulse (STIR WARP) shows abscess collections within the anterior surgical access route. Joint aspiration proved a low-grade infect with *Cutibacterium avidum*. The images were acquired at 1.5 T with following sequence parameters: STIR SEMAC CS—echo time (TE) 36 ms, repetition time (TR) 4220 ms, receiver bandwidth 500 Hz/pixel, inversion time (IT) 160 ms; STIR WARP—TE 31 ms, TR 4000 ms, receiver bandwidth 450 Hz/pixel, IT 150 ms

One drawback of advanced and hybrid metal artifact reduction techniques is the often long scan time. Two main approaches have been investigated to overcome this problem (Figs. 5 and 6). Compressed sensing (CS) has been used to accelerate SEMAC acquisition and demonstrated feasibility for imaging of total hip prosthesis and other anatomic regions, and for the differentiation between normal postoperative MRI findings and abnormal findings after total hip prosthesis [61–64]. The combination of CS and SEMAC has been shown to reduce acquisition times to 5–6 min for hip prostheses, allowing it to be incorporated easily into routine imaging protocols. At the same time, image quality is improved compared to standard SEMAC pulse sequences because of the increased number of slice-encoding steps. In addition, its high accuracy in detecting periprosthetic pathologies, such as infections, makes it a reliable sequence for assessing periprosthetic complications of large implants [50, 65, 66]. For MAVRIC imaging near metal implants, a short spectral calibration scan of about 35 s can be used for optimizing the number of spectral bins to minimize susceptibility effects and reduce scan time [17, 67]. For the combined MAVRIC-SL sequence, this calibration scan allowed to achieve scan times of 5–6 min for conventional acquisitions of hip prostheses compared to more than 8 min for MAVRIC imaging alone, and with an additional decrease of the repetition time an isotropic MAVRIC-SL was possible in 7:16 min with improved SNR [56] (Table 5).

**Table 5:** Sample protocol at 1.5-T MRI for patients with hip prostheses

Hip pulse sequences	Transverse STIR	Coronal STIR	Transverse T1	Coronal T2	Sagittal T1
MARS technique	–	SEMAC CS	–	–	–
Echo time (ms)	38	37	16	54	11
Repetition time (ms)	5040	5000	500	4480	593
Acquisition matrix (mm)	383 × 384	256 × 256	512 × 512	512 × 512	320 × 320
Slice thickness (mm)	7	3.5	6	4	4
Flip angle (°)	135	180	135	150	170
FOV (mm)	180 × 180	280 × 280	200 × 200	220 × 220	200 × 200
Bandwidth (Hz/pixel (kHz))	411	539	406	413	407
Inversion time (ms)	170	145	–	–	–
Averages	3	1	1	1	1
Turbo factor	9	13	3	18	3
Number of slices	30	28	33	24	34
Phase-encoding steps	419	359	619	571	576
Slice-encoding steps	–	12	–	–	–
Direction	A > > P	R > > L	R > > L	R > > L	H > > F
Acquisition time (min)	1:37	4:42	1:59	2:46	1:25

*MARS* metal artifact reduction sequences, *STIR* short tau inversion recovery, *CS* compressed sensing, *IM* intermediate weighted

Recently, the use of deep learning to reconstruct under-sampled MR data with the aim of accelerating image acquisition, reducing image noise, and improving image quality has attracted attention [68–70]. However, clinical applications of deep learning reconstruction for metal artifact reduction in MRI are still sparse and have yet to prove applicability in clinical routine [71].

## Summary

Advances in MRI around metal implants have significantly improved the visualization and diagnosis of implant-related abnormalities that cannot be assessed with other modalities. There are a variety of measures that can be taken to improve image quality around metal implants, but it is important to consider the material properties of the prosthesis, its geometry, and the clinical questions that need to be answered first, in order to use the correct sequences. Although metal artifacts are reduced at lower field strengths, today 1.5-T MRI is the preferred field strength for prosthesis imaging. For smaller, non-complex metal implants, basic metal artifact reduction techniques (increased receive bandwidth, reduced slice thickness, multi-echo spin-echo sequences, and STIR for fat suppression instead of frequency selective fat saturation) may be sufficient to obtain diagnostic images. However, advanced techniques such as CS-SEMAC and MAVRIC-SL should be used for imaging of large and complex metal implants such as joint prostheses, enabling a strong artifact reduction and reduced scan times. Finally, future developments in prosthesis composites and the application of evolving imaging techniques such as deep learning applications for metal artifact reduction may further improve imaging around orthopedic hardware.

## Data Availability

The data that support the findings of this study are not openly available due to reasons of sensitivity and are available from the corresponding author upon reasonable request.
